# From a national to an international journal: a new opportunity for the physiotherapy community

**DOI:** 10.1186/s40945-015-0004-y

**Published:** 2015-07-08

**Authors:** Marco Baccini, Marco Barbero, Roberto Gatti

**Affiliations:** 1 Editor-in-Chief of Archives of Physiotherapy, Motion Analysis Laboratory, Azienda Sanitaria di Firenze, Piero Palagi Hospital, Florence, Italy; 2 Rehabilitation Research Laboratory, Department of Business Economics, Health and Social Care, Manno, Switzerland; 3 President of the Italian Society of Physiotherapy, Laboratory of Analysis and Rehabilitation of Motor Function, Neuroscience Division, San Raffaele Hospital, Milan, Italy

**Keywords:** Virtual Reality Training, Physiotherapy Student, Reference Journal, Recent Cochrane Systematic Review, Physiotherapy Education

*Archives of Physiotherapy* is a new journal dedicated to the publication of scientific papers in the field of rehabilitation particularly physiotherapy. It is the continuation of the Italian Journal of Physiotherapy (*It J Physiother*), the scientific journal of the Italian Society of Physiotherapy (Società Italiana di Fisioterapia - SIF) that has been published by the Italian publisher Minerva Medica since 2011. The change goes far beyond the name of the journal, in that it also involves also the publisher, the type of publishing and the associations and institutions that acknowledge it as their official scientific journal. Before addressing the innovations, it is necessary to answer the question as to why one would launch a new journal in the field of physiotherapy, when the number of physiotherapy journals already exceeds far more than a hundred? The list available on the website of the International Society of Physiotherapy Journal Editors (ISPJE), (http://www.wcpt.org/ispje/membership) a network supported by the World Confederation for Physical Therapy (WCPT), includes 117 physiotherapy journals worldwide, with the Asia Western Pacific region having the highest number (67, 57.3 %) followed by Europe with 28 (23.9 %). The same question also presented itself at the time when the SIF board members decided to create the *It J Physiother.*

The answer to this question can be found in the determination to promote the scientific development of physiotherapy in countries where this discipline does not receive the credit it surely deserves, as for example in Italy, where physiotherapy is still considered the Cinderella of biomedical sciences. The SIF was in fact founded in 2010 with the declared purpose of advancing scientific activities within the physiotherapy field in Italy and supporting the dissemination of knowledge and clinical evidence-based practice (EBP) among Italian physiotherapists. Results have been encouraging, and the national congresses of the society, held yearly since its foundation, have given the opportunity to demonstrate the increasing level of Italian physiotherapists’ research activities [1].

Journals are generally considered to be the most important instrument for dissemination of research results and the promotion of EBP [2]. As for most of biomedical disciplines, however, high quality researches in physiotherapy are mostly found in journals which publish in English. Although the number of journals quoted in the ISPJE database is very high, only 44 of them (37.6 %) are published in the English language. In Europe there are only 9 journals in English, most of which (N = 7) are published in the United Kingdom. None of them are published in Italy nor in any other country in Southern and Eastern Europe, nor in many countries of Central Europe (e.g., Germany). Moreover, not one is the reference journal of the regional physiotherapy associations in these areas. Based on 2005 data retrieved in the European Region website of the World Confederation for Physical Therapy, it can be estimated that over 230,000 physiotherapists work in these countries (http://www.physio-europe.org/download.php?document=69&downloadarea=18), and therefore do not have direct or easy access to a scientific journal published in the English language. Since the language of publication is a crucial feature for the dissemination of findings, as demonstrated by the fact that systematic reviews frequently include only articles in English, there was an evident void before the launch of the SIF journal, that became the 10^th^ English language physiotherapy journal published in Europe.

Since its start in 2011, the journal adopted a double-blind peer reviewing model and favoured the quality, rather than the quantity, of published articles. In this regard, results have been encouraging. A survey on the first three years of activity [3] found that the proportion of published papers that are most relevant for EBP, i.e. systematic reviews and RCTs, was greater in the *It J Physiother* (44 %) than in other long-standing European physiotherapy journals. Results of one such RCT [4] were included in a recent Cochrane systematic review on the effectiveness of virtual reality training in stroke rehabilitation [5] – notably, no risk of bias was found by the Cochrane authors in this trial.

The number of submissions, and even more the quality of submitted articles, is a rather challenging matter for all novice journals, with limited visibility and lack of indexation in important biomedical databases. To enhance its visibility, visibility, *the It J Physiother* adopted a delayed open-access model: the journal's contents were accessible only to SIF members and to subscribers, but the full text was free in the case of articles over one year old. This model is used by many scientific journals and is a partial solution to the visibility problem. Many potential readers only saw the full text of *It J Physiother* contents one year after publication, unless they made the payment. The restricted access and the national perspective of the journal – inherent in the journal name itself – were, in the board's opinion, among the factors that were limiting the journal diffusion and development. Nonetheless, the last issues of the *It J Physiother* included a major number of articles from non-Italian authors, showing that it was beginning to attract researchers working in other European and even in non-European countries [3].

Early in 2014, the SIF board decided to bring about a radical advancement to the journal, in order to give it a more international perspective, increase its circulation and achieve indexing in important biomedical databases.

The desire of bringing the journal out of a merely national perspective explains the change of name. The new name – *Archives of Physiotherapy*, abbreviated to *Arch Physiother* – does not refer to a particular country or geographical area. However, the journal is primarily intended for physiotherapists working in Europe, in Southern and Eastern Europe in particular. It aims at becoming the future reference journal in these areas for professional associations, scientific societies and institutions concerned with the development of physiotherapy. A first step in this direction has been achieved by including the University of Applied Sciences and Arts of Southern Switzerland (Scuola Universitaria Professionale della Svizzera Italiana - SUPSI) in the project directed at the renewal of the SIF journal. In fact *Arch Physiother*, will be the official journal of this institution too.

Switzerland, like Italy, is a country where physiotherapy is taking big steps forward, with regards to both education and the promotion of scientific research. Between 2002 and 2006, the education for Swiss physiotherapy students was moved to a tertiary educational level and, in addition to SUPSI, physiotherapy is taught at present in three other Universities of Applied Sciences (UASs): Zurich University of Applied Sciences, Bern University of Applied Sciences and University of Applied Sciences and Arts of Western Switzerland. These institutions are therefore now responsible both for providing physiotherapy education and for boosting physiotherapy research with the aim of developing the profession. A first networking action bringing together UASs, hospitals, associations, policy makers, patients, clinicians and researchers has been promoted in order to increase the output of coherent research projects and to gain accountability within the Swiss healthcare system. Thanks to the UASs and the cooperation of various stakeholders, it has been possible to pinpoint research priorities for Swiss physiotherapists [6, 7].

Different strategies can be identified to enhance the visibility of the contribution of physiotherapists to the healthcare system. A recognized route is without doubt the dissemination of research results via peer-review publications. For this reason a new physiotherapy scientific journal supporting the profession's development was welcome and the SUPSI has joined the SIF in this challenging editorial project. Colleagues from the other UASs – along with internationally-renowned scholars in physiotherapy research – have accepted to enter the Editorial Board of *Arch Physiother* and hopefully others will do the same in the future.

The cooperation of the SUPSI in this project has allowed to undertake full open-access publishing – and, as a consequence, to choose the “open-access publisher” by definition, i.e. BioMed Central, signifying that all *Arch Physiother* articles are freely available immediately on publication. Indeed, with a full open-access model, the need of charging authors a fee to publish their articles is a major obstacle to receiving manuscript submissions, particularly for a new journal. The launch of a new open-access journal requires a solid business plan and a reliable budget to cover the costs of publication in the initial period, usually lasting several months, when articles are kept free for authors. Although the SIF is making an economical a contribution, the burden for the launch of *Arch Physiother* is being largely borne by the SUPSI which has obtained sufficient financing from the Thim van der Laan Foundation. Thanks to this foundation, authors will not need to pay an article-processing charge in the first four years of publication in *Arch Physiother*.

The open-access model is rather rare among physiotherapy journals, due likely (or at least in part) to the limited funding usually available to physiotherapy researchers. A notable exception is the Journal of Physiotherapy which, in January 2014, ushered in an innovative model where the journal content is free for readers and its publication is free for authors, by virtue of a strategic plan of the Australian Physiotherapy Association [8]. SIF and SUPSI have a similar plan for *Arch Physiother* and hope that in the future the cooperation of other partners and the involvement of physiotherapy associations and/or institutions in other European countries will allow to maintain this model in the coming years.

Open-access articles are more widely read [9] and are nearing the same scientific impact and quality as subscription journals, particularly in biomedicine [10]. However, the choice of an open-access model for *Arch Physiother* does not arise merely out of a need to ensure suitable visibility of the journal. Indeed, there is a growing awareness of some weakness in the traditional subscription model [11] as demonstrated by the thousands of new, full open-access journals launched in the last decade. Ethical issues, such as the promotion of public discussion and public deliberation, call into question the traditional restricted-access models and work in favour of more open approaches [12].

It has been shown that mortality rate among journals is quite high as is the birth rate [13]. Happily, four years after its birth the SIF journal is very much alive. Indeed, it is quite clear that it has grown and taken on new roots, thanks to which we are confident there will be even more growth in future years.
